# Sleep pattern in relation to recurrent osteoporotic fracture in the elderly

**DOI:** 10.3389/fpubh.2022.980352

**Published:** 2022-08-18

**Authors:** Likang Li, Haobin Zeng, Bo Zhang, Xu Xu, Maoshui Chen, Guowei Li

**Affiliations:** ^1^Center for Clinical Epidemiology and Methodology (CCEM), Guangdong Second Provincial General Hospital, Guangzhou, China; ^2^Department of Orthopedics No. 2 (Spinal Surgery), Guangdong Provincial Hospital of Chinese Medicine, Zhuhai, China; ^3^Department of Health Research Methods, Evidence, and Impact (HEI), McMaster University, Hamilton, OH, Canada

**Keywords:** Recurrent fracture, Elderly, Sleep pattern, osteoporotic fracture, Public Health

## Abstract

**Background:**

Previous studies assessed the relationship between individual sleep behavior and fracture risk, rather than taking into account the joint complexity of the sleep behaviors. We aimed to explore the association between sleep pattern and risk of imminent recurrent osteoporotic fracture in older hospitalized patients due to an index osteoporotic fracture, where sleep pattern was evaluated as a combination incorporating five common sleep behaviors (i.e., insomnia, snoring, nocturnal sleep duration, daytime napping, and midnight waking up).

**Methods:**

We used data from a prospective cohort study for analyses. Patients who aged not < 55 years and were admitted to the hospital due to an index osteoporotic fracture were recruited. Sleep pattern was grouped as healthy, intermediate, and poor pattern, based on the categorization of overall sleep scores. We used Cox proportional hazard models to explore sleep pattern in relation to imminent recurrent fracture.

**Results:**

We included a total of 185 elderly hospitalized patients for analyses with mean (± standard deviation) age = 71.5 ± 10.3 years and 87.0% female. During a mean follow-up of 14.7 months, there were 10 (5.4%) recurrent osteoporotic fractures observed. A significantly higher overall sleep score was found in patients with recurrent fractures when compared with those without fractures (3.20 vs. 2.36, *p* = 0.038). Both intermediate (*p* = 0.76) and poor sleep patterns (*p* = 0.093) were non–significantly associated with an elevated risk of fracture when compared with a healthy pattern. Per-one-increase in the overall sleep score was significantly related to an increased risk of fracture: hazard ratio = 1.60 (95% confidence interval: 1.00-−2.55) from the multivariable model.

**Conclusion:**

Per-one-increase in the overall sleep score was found to be significantly associated with a 60% higher risk of imminent recurrent osteoporotic fracture in the elderly, and intermediate and poor sleep patterns were non–significantly related to an increased risk of recurrent fracture. More high-quality evidence is required to further evaluate the relationship between the sleep pattern and the risk of recurrent osteoporotic fracture in the elderly.

## Introduction

As the population ages, the risk of osteoporotic fracture remained increasingly high worldwide, posing a significant challenge to the public health and global disease burden (1, 2). The risk of subsequent osteoporotic fracture in the elderly with an index osteoporotic fracture was substantially high, in which their subsequent fracture risk was two to six times higher than their controls who did not have a prior fracture (3). Despite an index fracture resulting in an elevated risk of subsequent fracture, there were <30% of postmenopausal women with an index fracture received anti-osteoporotic medications or prophylactic strategies (4). Therefore, more endeavors were required to enhance the management of osteoporosis and prevention of osteoporotic fracture in the elderly with an index fracture.

The relationship between sleep and osteoporotic fracture in the elderly had been extensively investigated in the literature (5–8). For instance, long sleep duration (hazard ratio = 1.26) and daily napping (hazard ratio = 1.33) were significantly associated with an increased risk of osteoporotic fracture in older women (7), while obstructive sleep apnea resulting in hypoxia was found to be associated with a 30–40% higher risk of fracture in older men (6). However, all the previous studies assessed the relationship between individual sleep behavior and fracture risk, rather than taking into account the joint and intrinsic complexity of the sleep behaviors. It was recommended to treat relevant sleep behaviors as an integrated pattern as appropriate because patients with one sleep characteristic were inclined to maintain another characteristic in general (9). Furthermore, while the previous studies focused on sleep in relation to the onset of osteoporotic fracture, evidence about the relationship between sleep and risk of imminent recurrent osteoporotic fracture in the elderly was limited and sparse. Therefore, in this study, we aimed to explore the association between sleep pattern and risk of imminent recurrent osteoporotic fracture in older patients who were hospitalized due to an index osteoporotic fracture, where sleep pattern was assessed as a combination incorporating five common sleep behaviors (i.e., insomnia, snoring, nocturnal sleep duration, daytime napping, and midnight waking up). Data from a Chinese prospective cohort study that enrolled elderly patients with hospital admission due to an osteoporotic fracture were used for analyses.

## Materials and methods

### Patients and setting

We recruited elderly patients from the Department of Orthopedics in a general hospital in Zhuhai, China initially from 2020 to September 2021 and extended the enrolment to December 2021 based on the consecutive sampling strategy. Given the official retirement age of 55 years for Chinese women and the high risk of osteoporotic fracture in postmenopausal women, we enrolled the patients aged not < 55 years. The patients were eligible for inclusion if they were admitted to the hospital due to an index osteoporotic fracture that was defined as any fragility fracture (but excluding fractures of face, fingers, and toes) and agreed to participate. The study procedure was similar to previous research conducted by the same authors (10).

Data were gathered *via* an in-person interview with trained research staff, laboratory measurements, and the hospital information system. We performed a follow-up (up to May 2022) by searching patients' medical records and calling them with the use of a telephone. All patients provided written informed consent before the study. This study was reviewed and approved by the Guangdong Second Provincial General Hospital Ethics Committee (No. 20190717-02-YXKXYJ-KT).

### Construction of integrated sleep pattern

The individual components and their scoring algorithm for the sleep pattern had been described according to Zeng et al. 2022 (10). In brief, we assigned 1 point to each of the five sleep behaviors if they were defined as *unhealthy* and 0 point if *healthy*, where healthy sleep behaviors included no frequent insomnia, no snoring, appropriate nocturnal sleep duration, appropriate daytime napping, and no frequent midnight waking up.

Patients were asked whether they had difficulty in falling and/or maintaining asleep in the previous 6 months, and if so, to document the *insomnia* frequency (rarely/never, sometimes, 1–3 times per week, and >4 times per week). Those with a response of 1–3 times or > 4 times per week were considered to have frequent insomnia. Regarding the information on *snoring*, patients were asked whether their family members ever told them or they themselves knew they had habitual snoring (yes/no). We documented the daily *nocturnal sleep duration* that patients had recently and categorized them as having appropriate sleep duration if they slept 7–9 h/day. Similarly, we asked whether patients owned habitual *daytime napping* and *midnight waking up* and recorded their duration of daytime napping and frequency of midnight waking up as appropriate. Appropriate daytime napping was defined as a daily napping duration of < 60 min, and no frequent midnight waking up was defined as a waking up frequency of ≤ 2 times each night.

Subsequently, we calculated an overall sleep score by adding up all the points from the five individual behaviors. The overall score had a minimum of 0 and a maximum of 5 points, with a larger score implying aggravated sleep. As recommended by the previous research (9, 11), we grouped patients as having a *healthy sleep pattern* if their overall sleep scores were 0 or 1 point, *intermediate pattern* if 2 or 3 points, and *poor pattern* if 4–5 points.

### Outcome assessment

The outcome was whether patients had imminent recurrent osteoporotic fractures after the index fracture during follow-up. We also collected data on the fracture site(s), the date of fracture, and whether the fracture led to hospitalization.

### Covariate information

Covariates of consideration were patients' baseline age, sex, smoking and drinking status, body mass index (BMI), lumbar spine T-score, comorbidities including diabetes mellitus and thyroid disease, use of anti-osteoporotic mediations before index fracture, and personal history of osteoporotic fracture in the previous 5 years. Their lumbar spine T-scores, as a measurement of bone mineral density, were evaluated by dual-energy X-ray absorptiometry (DXA) at L_1_-L_4_ using the same scanner (GE Prodigy, Madison, WI, United States).

### Statistical analysis

For descriptive statistics, we presented the results with mean ± standard deviation (SD) for continuous variables, and for categorical variables, we displayed the data with frequency and percentage. To compare differences between patients with and without imminent recurrent osteoporotic fracture, we used the Mann-Whitney *U* test for analysis of continuous variables and Fisher's exact test for categorical variables. Pie charts were used to display the proportions of different sleep patterns in patients with and without recurrent fractures. The Kaplan-Meier method was employed to graph failure curves for imminent recurrent osteoporotic fracture during follow-up.

We used Cox proportional hazard models to explore sleep pattern in relation to imminent recurrent fracture, with hazard ratios (HRs) and their corresponding 95% confidence intervals (CIs) reported. We first assessed the relationship between individual sleep behaviors and risk of recurrent fracture. Subsequently, we explored the association between sleep pattern and recurrent fracture risk, treating healthy sleep pattern as a reference. Results were presented from both univariable and multivariable (age- and sex-adjusted) models. As a supplemental analysis, we evaluated the relationship between overall sleep score and risk of recurrent fracture, taking overall sleep score as a continuous exposure variable. Results were shown as a per-one-point increase in the overall sleep score. Given the inconsistent findings on defining appropriate daytime napping, we performed another supplemental analysis by redefining appropriate daytime napping of < 30 min and rebuilding the sleep pattern. Spearman's correlation coefficient was then used to quantify the agreement between the original and the updated sleep pattern. Furthermore, we conducted another analysis by further adjusting for *T*-score in the multivariable model for sleep pattern in relation to the risk of recurrent fracture.

All analyses were performed with the use of STATA version 17 (StataCorp, College Station, TX) and SAS version 9.3 (SAS Institute, Inc., Cary, NC), with the significance level set at 0.05.

## Results

We included a total of 185 elderly hospitalized patients due to an index fracture for analyses (Table 1). They had a mean (± SD) age of 71.54 ± 10.32 years and a mean (± SD) BMI of 22.18 ± 3.40 kg/m^2^. The majority (87.03%) of included patients were females. There were 14.05% and 43.78% patients taking anti-osteoporotic medication before hospitalization and having a fracture history in the past 5 years, respectively. Their mean (± SD) of lumbar spine T-scores and overall sleep scores were−3.28 ± 1.70 and 2.41 ± 1.19, respectively. During a mean (± SD) follow-up of 14.73 ± 5.01 months, there were 10 (5.41%) recurrent osteoporotic fractures observed. The recurrent fractures included 1 thoracic and 9 lumbar spine fractures, all requiring hospitalization. Supplemental Figure 1 shows the Kaplan-Meier curve for the risk of recurrent fracture.

**Table 1:** Characteristic descriptions and comparisons between patients with and without imminent recurrent fracture.

**Characteristics**	**Total patients (*n* = 185)**	**Patients without recurrent fracture (*n* = 175)**	**Patients with recurrent fracture (*n* = 10)**	***P*-value**
Age: mean ± SD, in years	71.54 ± 10.32	71.28 ± 10.39	76.10 ± 8.27	0.14^1^
Females: *n* (%)	161 (87.03)	154 (88.00)	7 (70.00)	0.13^2^
Body mass index: mean ± SD, in kg/m^2^	22.18 ± 3.40	22.26 ± 3.42	20.72 ± 2.78	0.21^1^
Lumbar spine T-score: mean ± SD	−3.28 ± 1.70	−3.27 ± 1.74	−3.58 ± 0.69	0.73^1^
Current smoker: *n* (%)	18 (9.73)	16 (9.14)	2 (20.00)	0.25^2^
Current drinker: *n* (%)	10 (5.41)	10 (5.71)	0	-^3^
Taking anti-osteoporotic medication before hospitalization: *n* (%)	26 (14.05)	25 (14.29)	1 (10.00)	0.70^2^
Having an osteoporotic fracture in the past 5 years: *n* (%)	81 (43.78)	75 (42.86)	6 (60.00)	0.34^2^
With a diagnosis of diabetes mellitus: *n* (%)	32 (17.30)	31 (17.71)	1 (10.00)	0.53^2^
With a diagnosis of thyroid disease: *n* (%)	17 (9.19)	17 (9.71)	0	-^3^
Healthy sleep behaviors: *n* (%)
No snoring	145 (78.38)	139 (79.43)	6 (60.00)	0.23^2^
No frequent insomnia	100 (54.34)	96 (55.17)	4 (40.00)	0.52^2^
No frequent midnight waking up	138 (74.59)	132 (75.43)	6 (60.00)	0.28^2^
Appropriate nocturnal sleep duration	136 (73.51)	131 (74.86)	5 (50.00)	0.13^2^
Appropriate daytime napping	86 (46.49)	82 (46.86)	4 (40.00)	0.75^2^
Overall sleep score: mean ± SD	2.41 ± 1.19	2.36 ± 1.17	3.20 ± 1.32	0.038^1^

SD, standard deviation.

1 Based on Mann-Whitney U test.

2 Based on Fisher's exact test.

3 No test performed.

**Figure 1:**
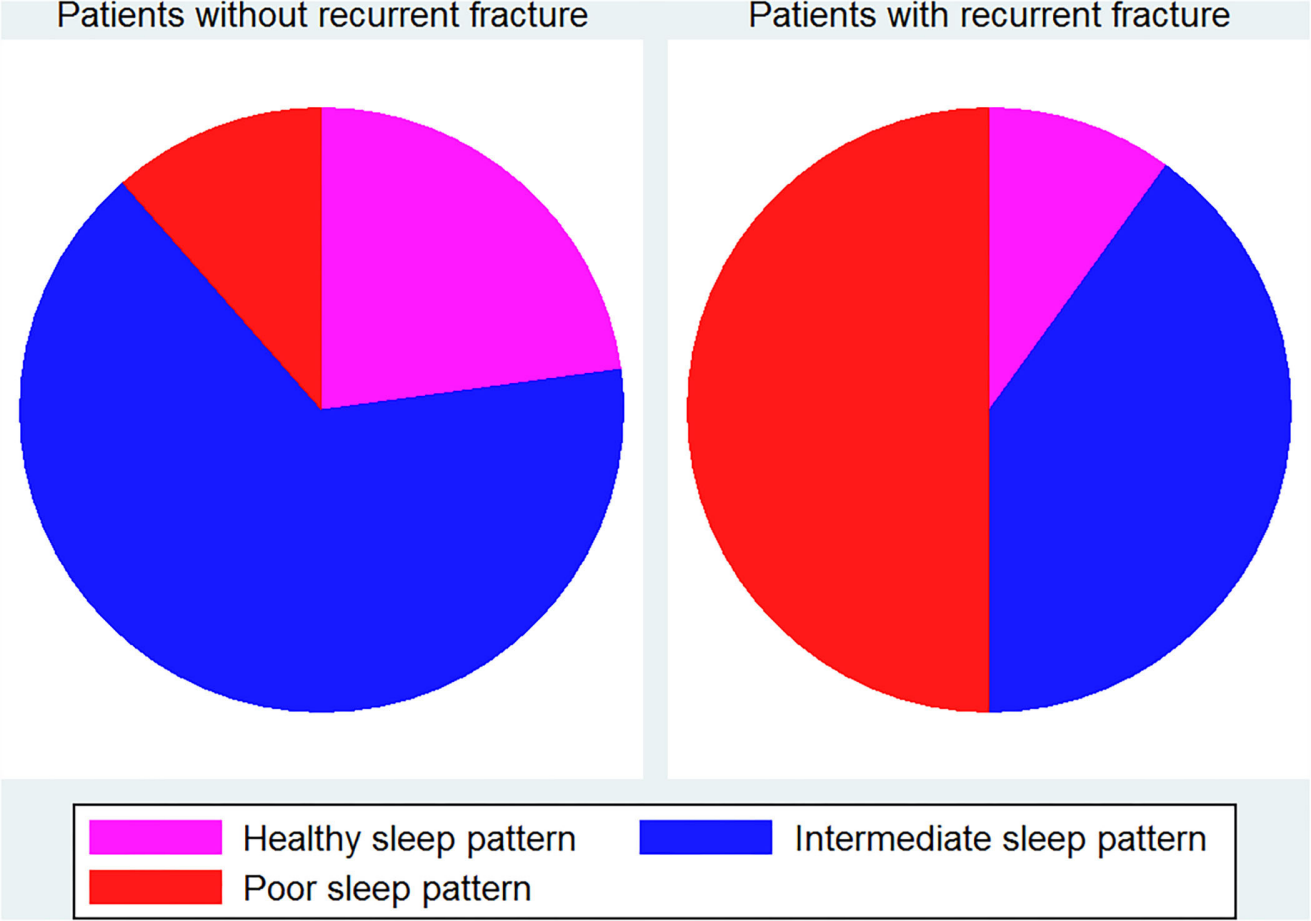
Pie charts of sleep pattern in patients with and without imminent recurrent fracture.

As shown in Table 1, no significant differences in age, female percentage, or BMI were found between patients with and without imminent recurrent osteoporotic fracture. Similarly, the two groups did not significantly differ in individual sleep behaviors; however, a significantly higher overall sleep score was found in patients with recurrent fractures when compared with those without fractures (3.20 vs. 2.36, *p* = 0.038). According to Supplemental Table 1 and Figure 1, there were 40 (22.86%), 111 (63.43%), and 24 (13.71%) patients having healthy, intermediate, and poor sleep patterns in patients without recurrent fracture, respectively, while the corresponding percentages were 1 (10%), 4 (40%), and 5 (50%) for patients with recurrent fracture. A significant difference in the sleep pattern proportions between patients with and without recurrent fractures was observed (*p* = 0.022).

The relationship between individual sleep behavior and risk of recurrent fracture is demonstrated in Supplemental Table 2. When compared with unhealthy sleep behavior, all the healthy sleep behaviors were related to an increased risk of recurrent fracture (HRs ranging from 1.23 to 2.70), albeit with non–significant results. According to Table 2, both intermediate and poor sleep patterns were non–significantly associated with elevated risk of recurrent fracture when compared with healthy pattern: HR = 1.24 (95% CI: 0.14-−11.15, *p* = 0.85) for intermediate and HR = 6.48 (95% CI: 0.76-−55.54, *p* = 0.088) from univariable model, and HR = 1.40 (95% CI: 0.16-−12.68, *p* = 0.76) for intermediate and HR = 6.45 (95% CI: 0.73-−56.78, *p* = 0.093) from multivariable model. Findings from the three supplemental analyses are shown in Table 3. Per-one-increase in the overall sleep score was significantly related to an increased risk of fracture: HR = 1.63 (95% CI: 1.01-−2.62) for univariable model and HR = 1.60 (95% CI: 1.00-−2.55) for multivariable model. The other two analyses yielded largely consistent results with findings from the main analysis.

**Table 2:** Relationship between sleep pattern and risk of imminent recurrent fracture.

**Sleep pattern^1^**	**Imminent recurrent fracture**	
	**Hazard ratio**	***P*-value**
	**(95% confidence interval)**	
***Model 1** ^**2**^*
Healthy	Ref	-
Intermediate	1.24 (0.14–11.15)	0.85
Poor	6.48 (0.76–55.54)	0.088
***Model 2** ^**3**^*
Healthy	Ref	-
Intermediate	1.40 (0.16–12.68)	0.76
Poor	6.45 (0.73–56.78)	0.093

1 There were 41 (22.16%), 115 (62.16%), and 29 (15.68%) patients with healthy, intermediate, and poor patterns, respectively.

2 Model for univariable analysis.

3 Model adjusted for age and sex.

**Table 3:** Supplemental analysis results for the relationship between sleep and risk of imminent recurrent fracture.

**Analysis**	**Imminent recurrent fracture**	
	**Hazard ratio (95% confidence interval)**	***P*-value**
***Taking sleep scores as continuous variable***		
Model 1^1^	1.63 (1.01–2.62)	0.044
Model 2^2^	1.60 (1.00–2.55)	0.050
***Using alternative definition for appropriate daytime napping** ^**3**^*		
Healthy	Ref	-
Intermediate	1.61 (0.20–13.11)	0.66
Poor	4.23 (0.38–49.97)	0.24
***Further adjusting for T-score***		
Healthy	Ref	-
Intermediate	1.60 (0.17–14.63)	0.68
Poor	7.60 (0.72–80.01)	0.091

1 Model for univariable analysis; data shown as a per-one-point increase in the sleep score.

2 Model adjusted for age and sex; data shown as a per-one-point increase in the sleep score.

3 Model for univariable analysis; there were 37 (20.00%), 119 (64.32%), and 29 (15.68%) patients with healthy, intermediate, and poor sleep patterns, respectively; Spearman's correlation coefficient with original sleep pattern was 0.76 (p < 0.001).

## Discussion

In this study, we evaluated the integrated sleep pattern in relation to the risk of imminent recurrent osteoporotic fracture in elderly patients with hospitalization due to an index fracture. A worsening overall sleep score was found in patients with recurrent fractures when compared with those without recurrent fractures (*p* = 0.038). Per-one-increase in the overall sleep score was significantly related to a 60% higher risk of recurrent fracture. The intermediate (*p* = 0.76) and poor sleep patterns (*p* = 0.093) were non–significantly associated with an increased risk of recurrent osteoporotic fracture when taking healthy sleep pattern as a reference.

We found an incidence of imminent recurrent osteoporotic fracture of > 5% in elderly patients, which was in line with previous studies (3, 12). Despite the progress of osteoporosis management and prophylactic interventions, the control of recurrent fractures remained suboptimal in elderly patients with index fractures (13, 14). Therefore, exploring sleep pattern as a lifestyle in relation to osteoporotic fracture in the elderly may provide some insights into identifying new approaches to enhanced prevention of recurrent fractures. Some physiopathological hypotheses, including inflammation, dysregulation of the sympathetic nervous system, and circadian rhythm disorder, had been found to explain sleep and the risk of osteoporotic fracture in the literature (15–17). We observed a significant relationship between overall sleep scores and the risk of recurrent fracture in the elderly (HR = 1.60, 95% CI: 1.00-−2.55 for per-one-increase in the overall score; Table 3), whereas the association between the fracture risk and the sleep pattern was not statistically significant (*p* > 0.05; Table 2). This was due to the categorization of a continuous variable leading to information loss in this study with a small sample size. In contrast, a significantly higher score in patients with recurrent fracture, in combination with the significant association between sleep scores and fracture risk, further suggested the link between the composite of individual sleep behaviors with fracture risk in the elderly.

Unlike previous research that assessed an individual sleep behavior, we used an integrated sleep pattern as a combination of common sleep behaviors for analyses. Specifically, the sleep pattern may be an integrated tool to evaluate both sleep time (including daytime napping and nocturnal sleep duration) and sleep quality (including midnight waking up, insomnia, and snoring). Individual sleep behavior may not substantially influence the recurrent fracture risk in the elderly, while an integrated sleep pattern would show a cumulative and prominent impact on the fracture risk. Therefore, sleep pattern could incorporate the combined and intrinsic influence of each individual sleep behavior, potentially making it a more appropriate measurement to reflect the comprehensive information on sleep than individual behaviors (10, 18). Daytime napping may be a compensation for inadequate nocturnal sleep duration, while insomnia may associate with frequent midnight waking up (9, 19). Although our results were in line with previous studies that an unhealthy behavior, including frequent insomnia, snoring, inappropriate sleep duration, inappropriate daytime napping, and frequent midnight waking up, was related to an increased risk of fracture (as shown in Supplemental Table 2), investigating one specific sleep behavior may thus fail to fully assess the impact of sleep on bone health and fracture prevention in the elderly.

In contrast, there were some scales intending to comprehensively appraise sleep in clinical practice, for instance, the Pittsburgh Sleep Quality Index. Of note, the requirement of clinical expertise, time, and efforts to complete these scales would preclude their wide application, especially when aiming to monitor sleep for public health. Thus, a simple sleep pattern based on common sleep behaviors and a straightforward scoring algorithm, if externally validated, may own the potential to help with prompt, accurate, and effective assessment of sleep health, especially in population-based studies. Nonetheless, more high-quality evidence is needed to further justify the reliability and validity of sleep pattern and their potential as a sleep evaluation tool.

Our observational study revealed a relationship between the sleep pattern and the risk of recurrent fracture from epidemiological data, which should be interpreted with caution. Confounding bias could not be fully addressed in a non–randomized study (20). For instance, a healthy sleep pattern in the elderly would in general relate to a low degree of frailty, other healthy lifestyles, and/or proactive prevention strategies, which may account for a decreased risk of recurrent fracture to an unknown extent. Unfortunately, we could not adjust for more covariates in models or perform further exploratory analyses, given the small number of fracture events. Therefore, our results were mainly hypothesis-generating, which required more research for exploration.

### Strengths and limitations

We targeted the imminent recurrent osteoporotic fracture in the elderly, given the aging population and substantially high risk of fracture recurrence within a short time after an index fracture for older patients. To the best of the authors' knowledge, there were no previous studies exploring sleep pattern in relation to recurrent fractures in the elderly. Findings from this study may generate some evidence for maintaining and improving sleep health to help with osteoporosis management, bone health, and fracture prevention in the elderly.

Several limitations existed in this study. One key limitation was the small sample size of recurrent fracture events, precluding our further analyses and exploration. We assessed the association between baseline sleep before an index fracture and risk of recurrent fracture, failing to take into consideration the change in or transition of sleep information after hospital discharge. Due to the data unavailability during follow-up, the impact of rehabilitation, medications, lifestyle change, and environmental adjustment on the association between sleep and recurrent fracture risk would remain unknown. The only additive algorithm was used to construct the sleep scores and patterns in this study. Furthermore, unquantified and unevaluated confounding factors may bias our findings, which could not be assessed unfortunately in this observational study. Some patients experiencing a recurrent osteoporotic fracture may not be aware of their fracture or receive a fracture diagnosis, which thus underestimated the recurrence of osteoporotic fracture. Moreover, our study sample consisted of hospitalized elderly patients with an index fracture; the generalizability of study results may therefore be weakened. Evidence of sleep pattern in relation to recurrent fractures for older dwellers would be needed to test the generalizability of our findings.

## Conclusion

In this study, we evaluated the integrated sleep pattern in relation to the risk of imminent recurrent osteoporotic fracture in elderly patients with hospitalization due to an index fracture. Per-one-increase in the overall sleep score was significantly associated with a 60% higher risk of recurrent fracture, and intermediate and poor sleep patterns were non–significantly related to an increased risk of recurrent osteoporotic fracture. More high-quality evidence was required to further evaluate the relationship between the sleep pattern and the risk of recurrent osteoporotic fracture in the elderly.

## Data availability statement

The original contributions presented in the study are included in the article/Supplementary material, further inquiries can be directed to the corresponding authors.

## Ethics statement

The studies involving human participants were reviewed and approved by the Guangdong Second Provincial General Hospital Ethics Committee (No. 20190717-02-YXKXYJ-KT). The patients/participants provided their written informed consent to participate in this study.

## Author contributions

LL, HZ, and GL contributed equally to this work. LL, HZ, MC, and GL designed the study and collected and analyzed the data. LL, HZ, and GL drafted the manuscript. BZ, XX, and MC provided professional help with manuscript writing and revisions. All authors read and approved the final manuscript. All authors contributed to the article and approved the submitted version.

## Funding

This study was funded by Science Foundation of Guangdong Second Provincial General Hospital (Grant no.: YY2018-002), the Science and Technology Program of Guangzhou (Grant no.: 202002030252), and the National Natural Science Foundation of China (Grant number: 82103906).

## Conflict of interest

The authors declare that the research was conducted in the absence of any commercial or financial relationships that could be construed as a potential conflict of interest.

## Publisher's note

All claims expressed in this article are solely those of the authors and do not necessarily represent those of their affiliated organizations, or those of the publisher, the editors and the reviewers. Any product that may be evaluated in this article, or claim that may be made by its manufacturer, is not guaranteed or endorsed by the publisher.
