# Supplementary material for: Sleep pattern in relation to recurrent osteoporotic fracture in the elderly
Source: Front Public Health. 2022 Aug 18;10:980352. doi: 10.3389/fpubh.2022.980352 (PMC9433782; doi:10.3389/fpubh.2022.980352)

Supplemental data

Supplemental Table 1. Sleep pattern groups in patients with and without imminent recurrent fracture*

Sleep pattern	Without recurrent fracture	With recurrent fracture
Healthy	40 (22.86)	1 (10.00)
Intermediate	111 (63.43)	4 (40.00)
Poor	24 (13.71)	5 (50.00)
Total	175	10

* Data shown as n (%); p-value was 0.022 based on Fisher’s exact test

Supplemental Table 2. Relationship between individual sleep characteristics and risk of imminent recurrent fracture

Sleep characteristic	Risk of imminent recurrent fracture[1]	
	**HR (95% CI)**	**P-value**
**Snoring**		
No (n = 145, 78.38%)	Ref	-
Yes (n = 40, 21.62%)	2.37 (0.67 - 8.39)	0.18
**Frequent insomnia**		
No (n = 100, 54.34%)	Ref	-
Yes (n = 85, 45.66%)	1.70 (0.48 - 6.03)	0.41
**Frequent midnight waking up**		
No (n = 138, 74.59%)	Ref	-
Yes (n = 47, 25.41%)	1.73 (0.49 - 6.14)	0.40
**Appropriate nocturnal sleep duration**		
Yes (n = 136, 73.51%)	Ref	-
No (n = 49, 26.49%)	2.70 (0.78 - 9.35)	0.12
**Appropriate daytime napping**		
Yes (n = 86, 46.49%)	Ref	-
No (n = 99, 53.51%)	1.23 (0.35 - 4.34)	0.76

HR = hazard ratio; CI = confidence interval

[1] Results from univariable model

Supplemental Figure 1. Kaplan-Meier curve for the risk of imminent recurrent osteoporotic fracture in the elderly

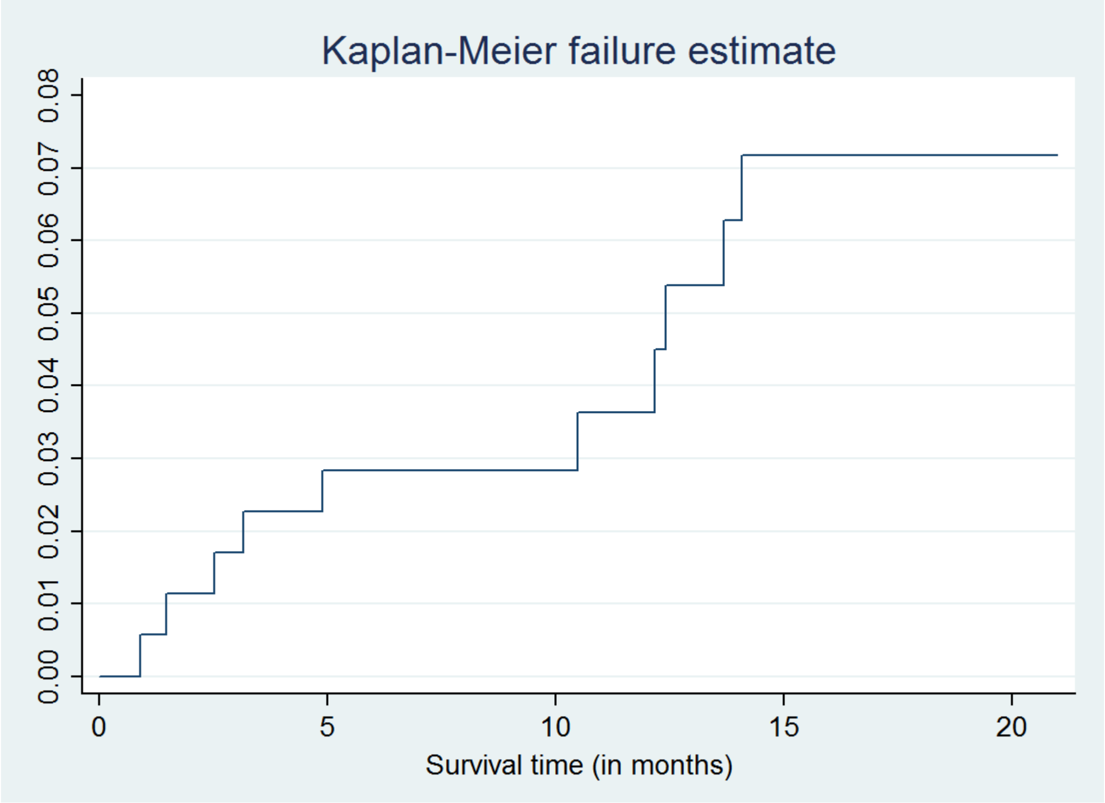

Supplement: Supplementary file 1 [file Data_Sheet_1.pdf]
